# Transitional vascular anomaly of a persistent medial procephalic vein causing obstructive hydrocephalus and intracranial haemorrhage

**DOI:** 10.1259/bjrcr.20220064

**Published:** 2022-11-01

**Authors:** Masis Isikbay, Kazim Narsinh, Michael Caton, Matthew Amans

**Affiliations:** 1 Department of Radiology and Biomedical Imaging, University of California San Francisco, 505 Parnassus Ave, M-396, San Francisco, CA, United States

## Abstract

We report a case of obstructive hydrocephalus caused by a transitional (shunting) developmental venous anomaly not previously reported in the literature. Both thalami in this patient drain into a midline vein in the floor of the third ventricle that crosses the cerebral aqueduct and exerts mass effect. While this patient’s hydrocephalus was managed by a ventriculoperitoneal shunt catheter, their hospital course was complicated by a spontaneous intraparenchymal bleed of the left thalamus thought to be caused by their vascular malformation. Given the risk of venous infarcts, this transitional venous anomaly could not be treated safely.

## Introduction

Developmental venous anomalies (DVAs) are the most common cerebral vascular abnormality^1^ resulting from variations in venous development. A subtype of DVAs are transitional venous anomalies (TVAs) which demonstrate features similar to an arteriovenous malformation (AVM), namely early arteriovenous drainage but without a parenchymal nidus.^2^ DVAs and TVAs are often the sole venous drainage of their associated parenchyma. While rare occlusion of the DVA/TVA can cause venous congestion, and potentially life-threatening intraparenchymal haemorrhage.^3,4^ Another rare complication of a TVA is obstructive hydrocephalus caused by the TVA compressing/coursing within and occluding the cerebral aqueduct.^5^

Our presented case is unique because the patient experienced both rare complications from the same the same TVA. Their malformation also drew attention to an undescribed anatomical variant with a draining vein that courses along the floor of the third ventricle. Given this vein’s role in draining both thalami, the risk of treatment was too great and medical management was recommended as embolisation of this vessel would likely cause bilateral thalamic infarcts in a pattern similar to deep cerebral venous thrombosis.^6–9^

## Case presentation

**Figure 1.**
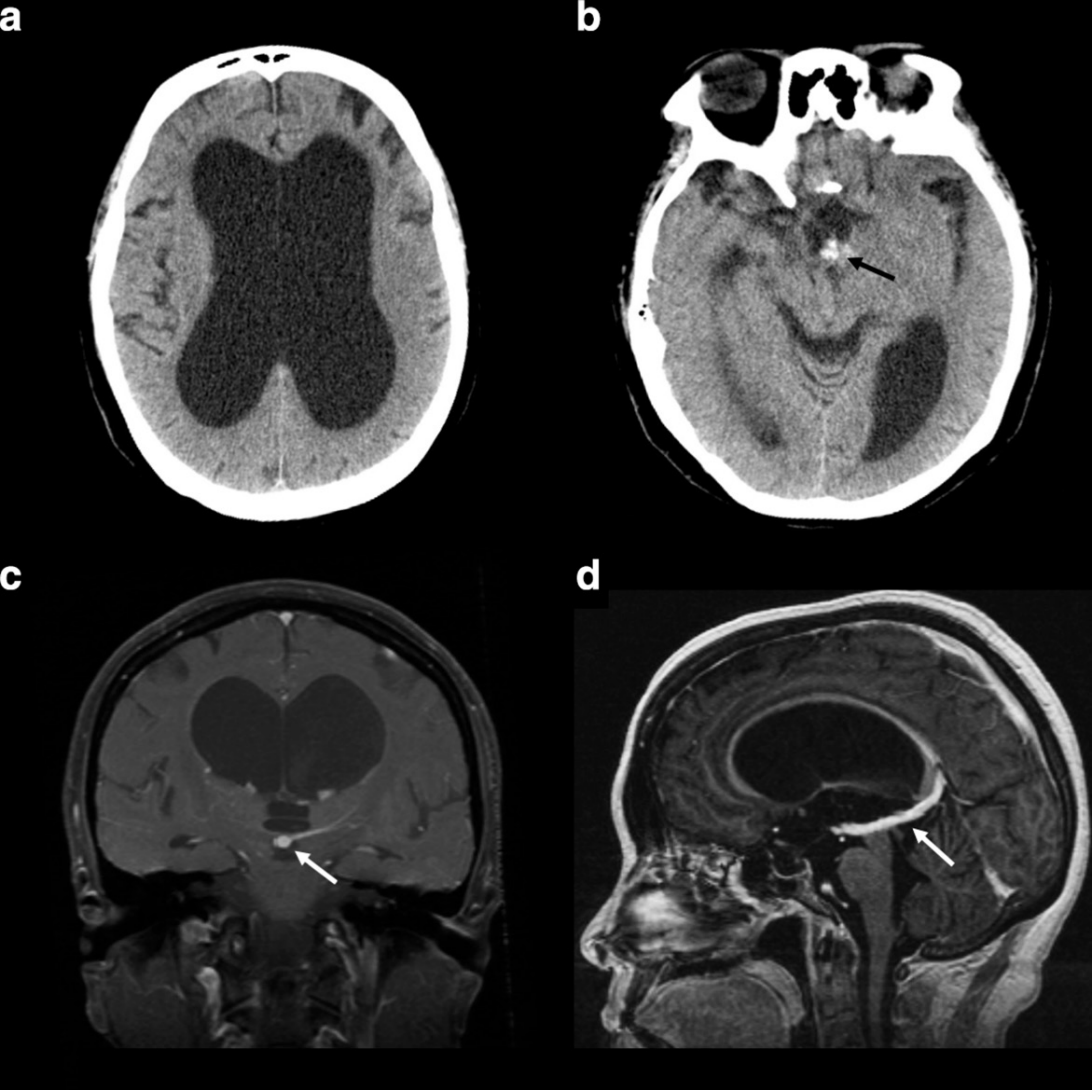
Non-contrast head CT scans from the patient’s initial presentation demonstrates hydrocephalus (panel A) as well as blood products in the suprasellar cistern (panel B, black arrow). A follow-up MRI with post contrast *T*_1_ weighted imaging reveals the presence of a dilated central vein (C, D white arrows) with compresses the cerebral aqueduct and is thought to be the cause of the patient’s hydrocephalus.

A female in her 50s with no significant past medical history presented to their local emergency room with complaints of a persistent headache and bilateral leg weakness that began 1 week ago. She had no history of trauma or prior history of any neurological symptoms. Her physical exam was only notable for hyperreflexia in the bilateral lower extremities. A non-contrast CT scan of the brain demonstrated marked hydrocephalus (Figure 1A) and blood products were seen in the suprasellar cistern adjacent to the third ventricle (Figure 1B). The patient was admitted to the medicine service with neurosurgical consultation to manage the hydrocephalus and investigate the cause of her intracranial haemorrhage.

An MRI was performed and identified a vascular malformation adjacent to the third ventricle compressing the cerebral aqueduct. This finding was suspicions for an AVM and/or a DVA/TVA given the presence of a prominent central vein (Figure 1C and D). No AVM was appreciated on a subsequent catheter directed cerebral angiogram.

The patient’s neurological status improved, they were discharged a few days later. Because of persistent ataxia and hydrocephalus, weeks after the initial presentation a ventriculoperitoneal (VP) shunt was placed without complication. Following this procedure, the patient’s headache and ataxia completely resolved.

Over the course of the following 15 years, the patient has serial imaging that demonstrated no change in her hydrocephalus. During this time, the patient had multiple presentations to the emergency department for falls in the setting of intermittent generalised weakness. Some of these episodes were associated with significant trauma which included intracranial haemorrhage as well as upper extremity fractures.

**Figure 2.**
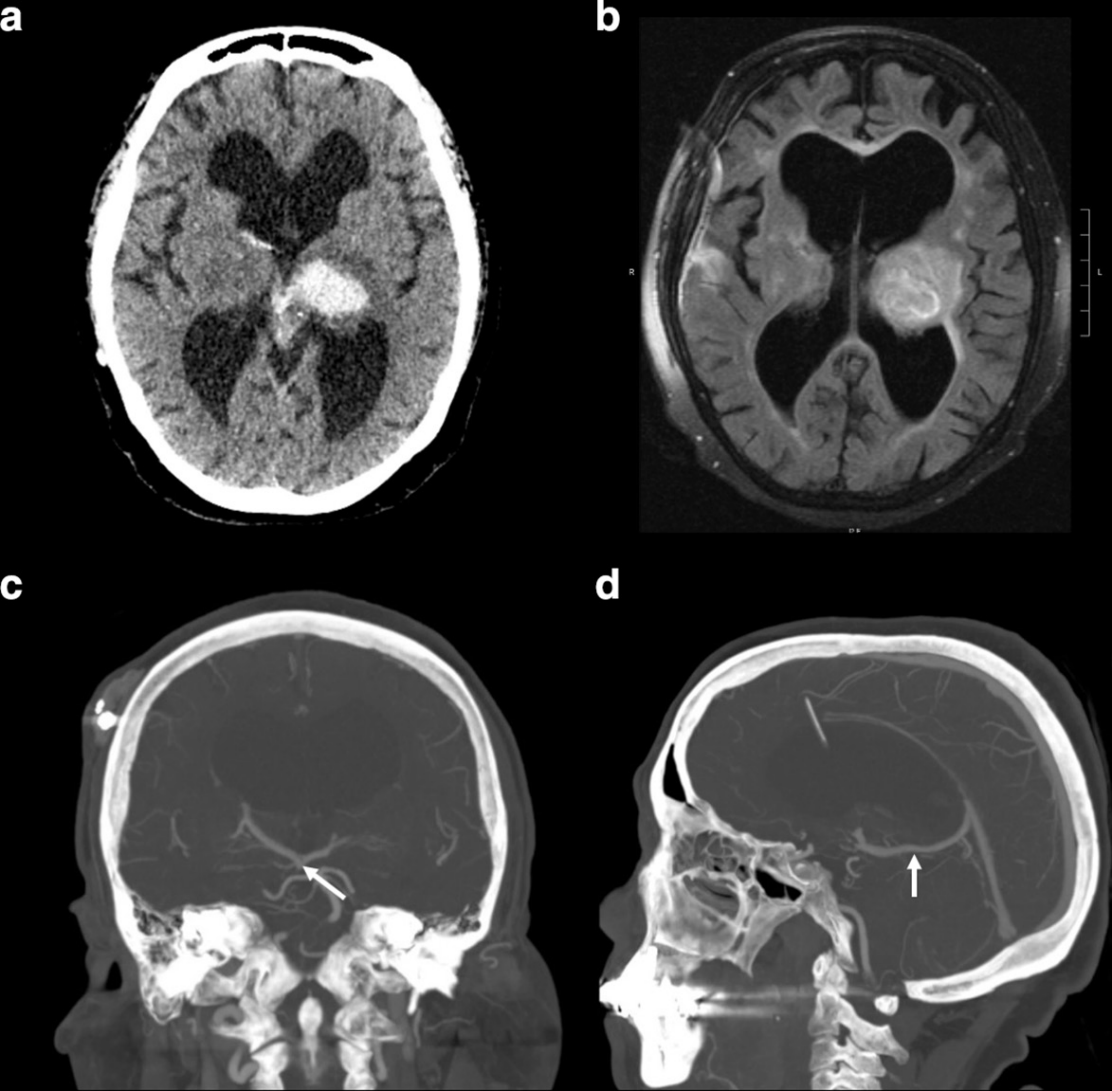
After the patient’s stroke activation, a non-contrast head CT scan is performed which reveals a left thalamic bleed (panel A). A follow-up MRI with *T*_2_ weighted FLAIR imaging demonstrates significant signal around the left thalamus (panel B) suggestive of edema. CTA imaging re-demonstrates the dilated central vein which compresses the cerebral aqueduct (panels C, D white arrows). TA, CT angiography.

Following these episodes, the patient presented to the hospital as a stroke activation with right-sided hemiparesis, leftward gaze deviation, and aphasia. Vital signs were notable for a blood pressure of 163/94 and a heart rate of 77. Stroke scale was initially 20 and worsened to 26. Glasgow coma scale (GCS) was 12. A CTA of the head showed a large left thalamic intraparenchymal haemorrhage (Figure 2A) and a prominent central vein compressing the cerebral aqueduct was again seen (Figure 2C and D). The patient was admitted to the neurology ICU for stabilisation where she was normotensive. A head MRI demonstrated significant vasogenic oedema surrounding the left thalamic bleed suspicious for a venous aetiology (Figure 2B). She was stabilised and discharged 3 days later to a skilled nursing facility with persistent aphasia and right-sided weakness.

**Figure 3.**
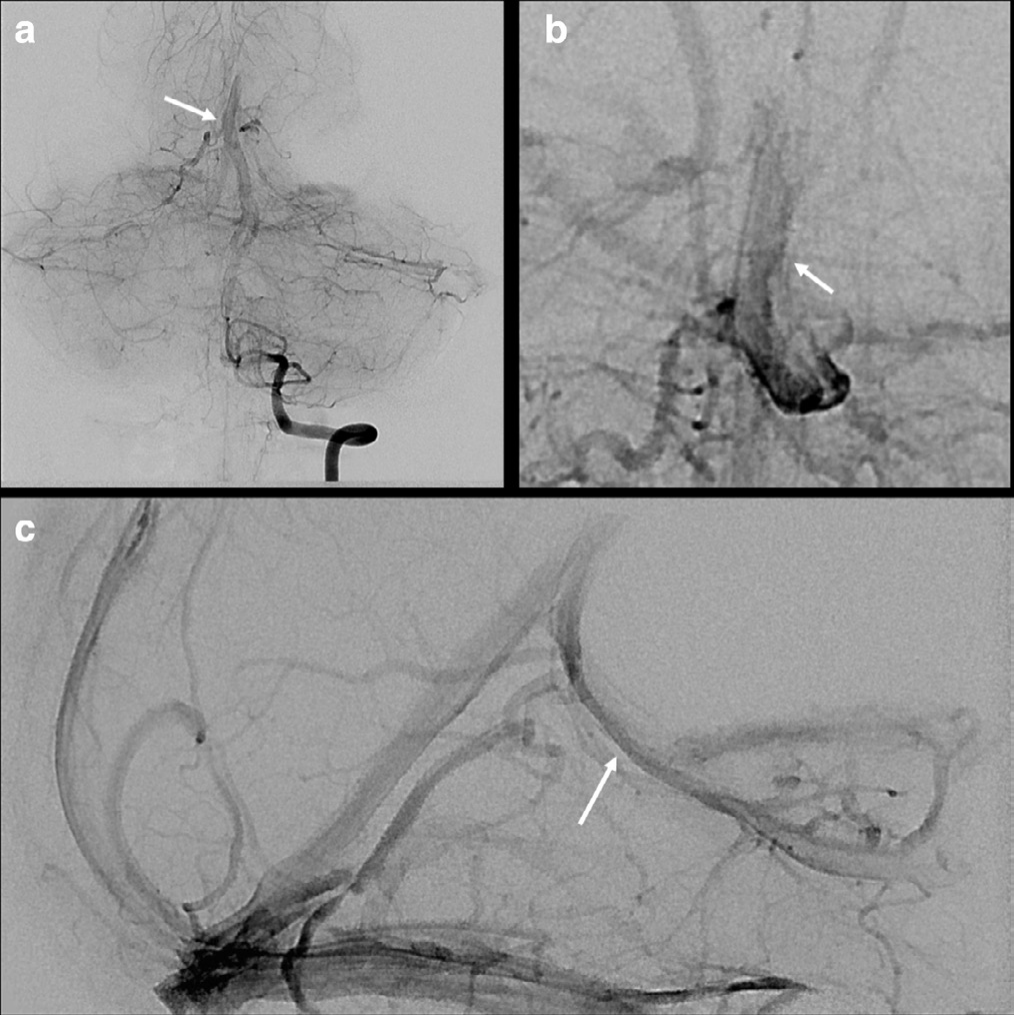
Repeat catheter directed cerebral angiography shows early venous drainage via a dilated central vein (panel A, white arrow) which runs along the floor of the third ventricle. This represents the patient’s transitional developmental vascular abnormality. This vessel is appreciated on anterior to posterior (panel B) and lateral (panel C) projections (highlighted with the white arrows).

This patent was then referred to our centre for further evaluation. Our angiogram showed early venous drainage (Figure 3A) into a TVA involving both thalami and coursing into a midline vein in the floor of the third ventricle (Figure 3B and C). The vessel exited the third ventricle through the cerebral aqueduct where it occludes the flow of CSF causing non-communicating hydrocephalus.

Despite the patient’s clinical course, this vascular lesion was not amenable to treatment/embolisation as this would likely cause venous infarcts, and the side-effects of infracting the thalami and basal ganglia clearly outweighed the risks of re-bleeding. This patient as managed conservatively with blood pressure control using losartan which was successful in keeping her normotensive. In the time since the most recent cerebral angiogram, there have not been any relevant clinical changes.

## Discussion

This case demonstrates multiple features of the patient’s transitional venous abnormality which are noteworthy. While in most cases TVAs are considered to be benign, in rare cases they can cause intracranial haemorrhage^3,4^ they can sometimes cause obstructive hydrocephalus.^5^ Our presented case is unique because both complications were experienced by the patient (which has not previously been reported). These events led to long-term neurological deficits notable for aphasia and right-sided weakness.

**Figure 4.**
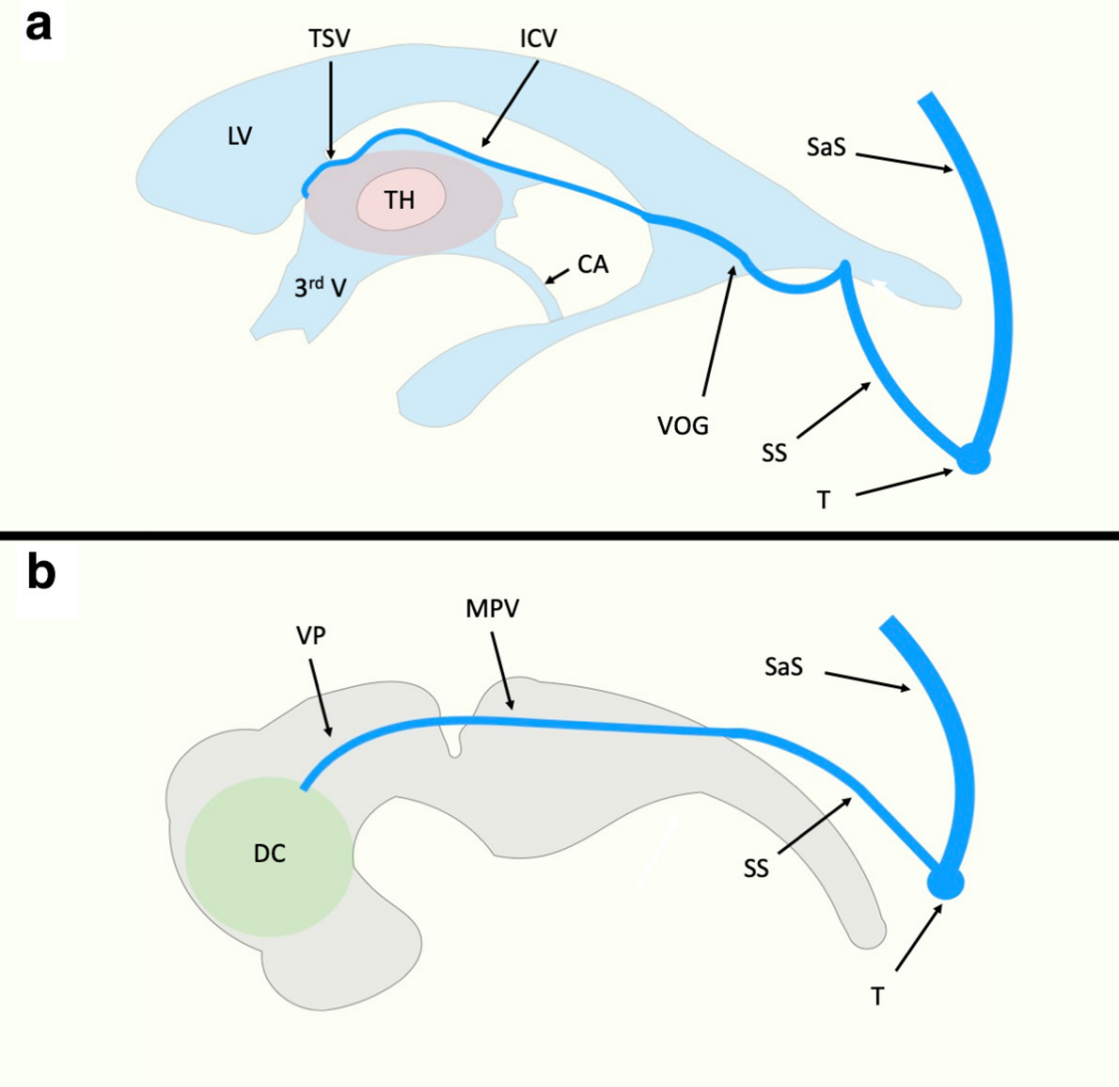
Original author created figure demonstrating key anatomy. TSV drain into the ICVs which run along the roof of the third ventricle. The ICV anastomose to the VOG which ultimately drains into the torcular heterophili (T) via the SS. In the embryologic stage (panel B), a venous plexus is responsible for draining important regions of the growing central nervous system such as the diencephalon (D). This plexus is predominantly drained by the PMV which drains into the developing SaS. The PMV becomes the VOG and precedes the development of the ICVs. ICV, internal cerebral vein; PMV, median prosencephalic vein; SaS, sagittal sinus; SS, straight sinus; TV, thalamostriate vein; VOG, vein of Galen

Furthermore, this patient’s TVA was particularly rare given that it involved a central draining vein which has not previously been characterised in this type of clinical context. Given a review of the expected embryological development of the cerebral veins,^10,11^ this vessel most likely is a persistent median prosencephalic vein (MPV)/vein of Markowski^12^ mimicking anatomy that has been previously reported.^13^ During development, this vessel is a precursor to the vein of Galen, and is present in development prior to the formation of the internal cerebral veins,^13–15^ which is shown in Figure 4A and B.

**Figure 5.**
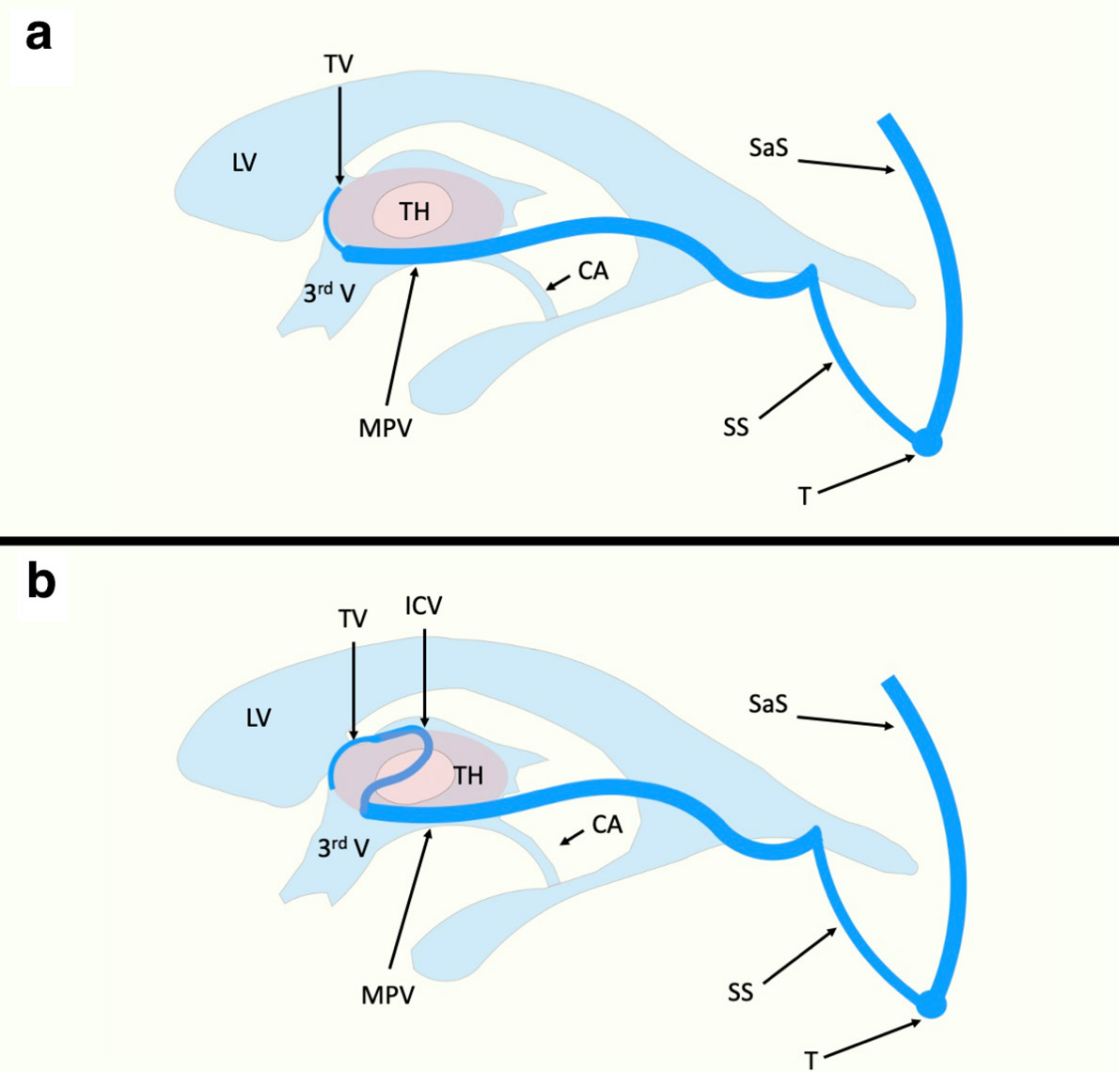
Original author created figure demonstrating key anatomy. In the presented patient a persistent and dilated PMV runs along the floor of the third ventricle and obstructs the CA. Drainage of the thalami differed by laterality: On the right (Panel A), the thalamic veins drain directly into the PMV. On the left (Panel B) the thalamic veins drain into an internal cerebral vein, which in turn connects to the PMV. No true vein of Galen is present and the PMV anastamoses directly to the SS. CA, cerebral aqueduct; PMV, median prosencephalic vein; SS, straight sinus.

In our presented case, the patient’s thalamic venous drainage is unique and even varies based upon laterality. There are thalamic veins bilaterally; on the right, this vein drains directly into the MPV, and there is no internal cerebral vein (Figure 5A). On the left, the thalamic vein first drains into an internal cerebral vein (Figure 5B). No vein of Galen is present consistent with the expected developmental pathway. Given the above anatomy, despite the complications experienced by the patient, therapy options were limited. While the hydrocephalus was well managed by the placed VP shunt, the persistent median prosencephalic vein could not be safely embolised. Treatment of this vessel would cause loss of appropriate thalamic venous drainage, and would result in bilateral thalamic infarcts in a pattern well documented by cerebral venous thrombosis.

## Conclusion

While DVAs/TVAs are among some of the most common cerebral vascular abnormalities, in rare instances they can be responsible for causing life-threatening complications. We present a case where a TVA involving a persistent medial procephalic vein is responsible for causing both obstructive hydrocephalus and haemorrhagic stroke. While the combination of these complications from a previously undescribed vascular malformation is exceedingly rare, it raises awareness regarding the nuances of treatment options. Given its sole responsibility for the venous drainage of both thalami, the embolisation of this TVA would cause venous infracts, making medical management and surveillance the only viable therapeutic strategy.

## Learning points

- While DVAs/TVAs are common and usually are benign, they can cause serious complications which can result in long-term neurological deficits.
- While rare, DVAs/TVAs can cause obstructive hydrocephalus by exerting mass effect on structures such as the cerebral aqueduct.
- TVAs can alter venous drainage pathways in the brain, which can result in intracranial haemorrhage secondary to venous congestion.
- The median prosencephalic vein is an important precursor in the development of the cerebral venous system and is normally absent after the internal cerebral veins/vein of Galen forms.
- Venous malformations that are responsible for supplying venous drainage to the thalamus cannot safely be embolised given the risk of thalamic infarcts.
